# A Comprehensive Review on Date (*Phoenix dactylifera* L.) Syrup: Functional Properties, Innovative Extraction Approaches, and Main Applications in the Food Industry

**DOI:** 10.3390/foods15132400

**Published:** 2026-07-07

**Authors:** Younes Noutfia, Ewa Ropelewska, Sara Silva, Monika Mieszczakowska-Frąc

**Affiliations:** 1 Research Unit on Nuclear Techniques, Environment, and Quality, National Institute of Agricultural Research, Tangier 90000, Morocco; 2 Fruit and Vegetable Storage and Processing Department, The National Institute of Horticultural Research, Konstytucji 3 Maja 1/3, 96-100 Skierniewice, Poland; ewa.ropelewska@inhort.pl (E.R.); monika.mieszczakowska@inhort.pl (M.M.-F.); 3 Universidade Católica Portuguesa, CBQF—Centro de Biotecnologia e Química Fina-Laboratório Associado, Escola Superior de Biotecnologia, Rua Diogo Botelho 1327, 4169-005 Porto, Portugal; snsilva@ucp.pt

**Keywords:** dibs, concentrated juice, polyphenols, health, processing, sugar substitution, natural sweeteners, by-products valorisation, eco-friendly extraction

## Abstract

Date syrup is a product processed from date (*Phoenix dactylifera* L.) flesh through a multi-unit operation process. This nutritious liquid is rich in carbohydrates, dietary fibres, polyphenols, minerals, and vitamins, and may provide functional and bioactive compounds with potential health-promoting properties. In this review, a summary of current literature related to the technological aspects of date syrup production was given with a focus on traditional, conventional, and novel extraction methods. Further, a systematic analysis of available data describing the compositional quality of date syrup based on biochemical, functional, antioxidant, and pro-healthy properties was highlighted. Finally, the potential applications of date-based syrup in several fields of food industry were discussed by highlighting the importance of using date syrup as an emerging alternative for sugar substitution in numerous food products and formulations.

## 1. Introduction

Date palm (*Phoenix dactylifera* L.) is one of the most valuable domesticated fruit trees because of its ritual significance in human societies, health benefits, productive capacity, and the range of subsistence products obtained from its fruits and other parts of the large palm [1]. It is widely known as a versatile crop that holds historical significance and promising future progress. The size of the date crop is increasing rapidly in the world, and the production of date fruits was about 9.66 million tonnes in 2023 [2]. Date is a stone fruit characterised by a fleshy mesocarp and papery endocarp with large variability among cultivars estimated to more than 1500 cultivars around the world [3]. Recently, more attention has been given to this “blessed tree” and its fruit, given the numerous nutritional, therapeutic, and pharmacological applications explored in several studies. In addition, emerging data support the notion that intake of date fruit and extracts can be a useful component of a healthy lifestyle for those seeking beneficial effects on vascular health [4,5,6,7]. Also, it has been found that date fruits provide an appreciable amount of energy required for the human body and also act as an effective therapeutic agent against several diseases [6].

Moreover, date fruits are rich with a multitude bioactive and functional compounds such as polyphenols, dietary fibres, carotenoids, vitamins, amino acids, carbohydrates and mineral elements, making it one of the most nourishing natural foods [8,9]. The principal component of date fruit flesh is sugars, ranging from 60 to 80% depending on two key factors: harvesting stage (generally the high concentration of sugars is recorded at the ‘Tamar’ stage), cultivar, and category such as soft, semi-soft, or dry date fruit [4,10,11]. Usually, soft date cultivars are characterised by high concentrations of glucose and fructose (invert sugars) with a small amount or no saccharose, inversely to dried date cultivars dominated by this complex sugar (saccharose). Thus, another classification of date was suggested based on “sugars type” and outlined three classes: (1) invert sugar dates such as ‘Mejhoul’, ‘Barhi’, ‘Saidi’, etc., (2) mixed sugar cultivars such as ‘Khadrawy’, ‘Zahidi’, etc., and (3) the cane sugar class involving mainly the ‘Deglet Noor’ cultivar [11]. This richness in terms of sugar categories, sucrose, glucose, and fructose and their relative high concentrations make date fruit a promising source and potential raw material for food processing. Date syrup is one of the most known processed by-products of date flesh and it is characterised by a great amount of carbohydrates/sugars, as well as minerals and vitamins. This concentrated nutritious liquid is produced according to a multi-unit operation process with extraction as the main and critical step [12]. After extraction and purification adjustments, the final product (date syrup) can be consumed directly or incorporated into other food formulations in the objective to improve the nutritional and the healthy score of these formulations.

To bring comprehensive insights around this date by-product, this review paper aims to explore all aspects related to “date palm syrup” based on a holistic literature analysis.

This review was conducted as a narrative literature review with a structured research approach. Relevant publications were identified through searches on Google Scholar, Scopus, and Web of Science. The main search terms included “date”, “*Phoenix dactylifera* L.”, “extraction”, “functional compounds”, “health”, and “food formulation” used individually and in various combinations. The literature search covered published studies between 2016 and 2026 with a few other relevant investigations. Peer-reviewed original research articles and relevant review papers were included and articles lacking sufficient scientific relevance were excluded. Thus, this review is structured on three coherent and complementary pillars that address the following:

- Traditional, current, and innovative techniques of date syrup processing and extraction (Section 1);
- Compositional and functional characteristics with a focus on antioxidant, antibacterial. and health benefits (Section 2);
- Main technological–industrial applications of date syrup in the food industry (Section 3).

## 2. Traditional and Novel Extraction Techniques of Date Syrup

**Figure 1:**
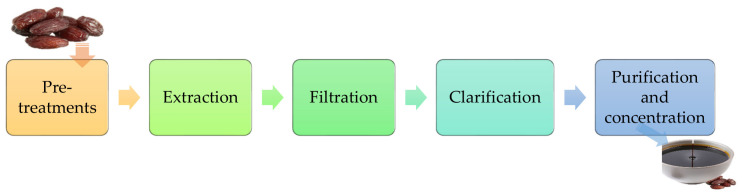
Classical process of date syrup production.

**Figure 2:**
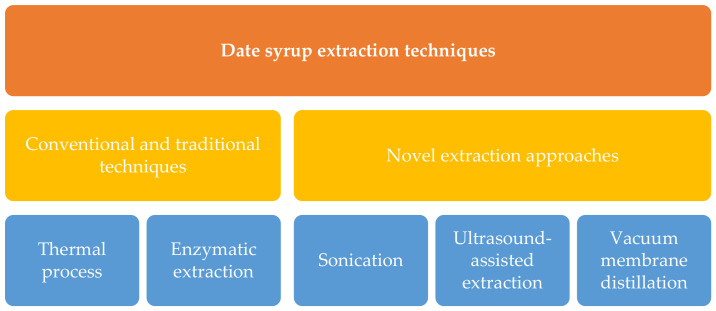
Illustration of different extraction techniques of date syrup production.

Regardless of the extraction technique, the technological process of date syrup production follows the generic scheme [12] shown in Figure 1. Generally, pre-treatments consisted of washing, pitting, and cutting date fruit into small pieces to maximise the yield of extraction. Once the pulp is obtained, an adequate extraction technique is applied (Figure 2) according to facility capacity in terms of equipment and knowledge of processing.

The extracted syrup is then filtered to remove remaining impurities and colloidal substances and subsequently submitted to clarification, purification, and concentration in the objective of improving viscosity and reducing turbidity, as well as obtaining a date palm syrup with a high level of sugars and bioactive compounds [13,14,15].

To ensure a high final quality of the produced syrup, the “extraction” is usually linked to “clarification” and in several cases the two processes are combined into one continuous process. Accordingly, the attenuation/elimination of dark colour phenomena is guaranteed by employing cation–anion exchanges, ion exchange adsorption, and selected membrane methods [16,17,18,19]. Moreover, date syrup extraction was known as one of the oldest techniques in the processing and valorisation of date fruit [12,16]. This process can be naturally produced while the fruit is stored under hot conditions or artificially carried out using various approaches as reported in Figure 2.

### 2.1. Traditional Extraction of Date Syrup in Palm Oases

Since a long time ago, date syrup was produced in several palm oases in Arabic countries and took different forms according to the producing area (Table 1). The traditional preparation of date syrup consisted of cleaning the date fruit, manual pitting, and cooking pitted (in some cases with seeds) date fruit in boiled water [20]. Then, the mixture “water-date” is filtered through fine fabric or some polypropylene woven bags [21]. The obtained liquid is heated a second time in order to give the date syrup its final characteristics in terms of viscosity and sugar content. However, this step remained subjective and traditionally determined. The end point is checked by dipping a few drops of the concentrated liquid on the sand [21]:

- If these droplets easily penetrate the sand, the concentration of syrup is qualified as not yet having been reached;
- If the droplets constitute small semi-solid balls on the surface of the sand, the expected consistency/concentration of the syrup is achieved.

**Table 1:** Common names of date syrup in some autochthonous areas of production.

Region of Production	Morocco	North Africa	Middle East	Iraq
Common name	Tahlawt	Rob	Dibs	Assal/Honey
Reference	[16]	[20]	[22]	[23]

In traditional extraction, the process parameters (especially temperature, time, Brix of final syrup) are not defined and affect the physicochemical and organoleptic properties of date syrup. Hence, an improved manufacturing diagram was suggested to investigate the effect of some modifications in traditional extraction on the sensory and physicochemical attributes of date syrup obtained from three Tunisian cultivars [20]. This improved extraction, based on defined temperature and extraction time, confirmed its positive effect on carbohydrates, lipid and protein fractions, polyphenols, and minerals.

Overall, the traditional extraction of date syrup is based on thermal processes and is often associated with the degradation of several bioactive and nutritional compounds. In addition, this technique negatively influences the syrup colour [12] but is still used to create the traditional date-based syrup used widely in the traditional treatment of several diseases such anaemia [24].

### 2.2. Enzymatic Extraction

As mentioned in traditional extraction, the produced date syrup is characterised by high turbidity and brown-dark colour caused by thermal operations and concentrations of soluble compounds. In particular, coloured agents such as melanoidins, melanins, and caramels caused the unsuitable colour of date syrup and juice, while pectin and polyphenol compounds are the main turbidity-causing substances [19,25]. To alleviate these phenomena, enzymatic treatments were investigated to improve the quality attributes and commercial value of date syrup.

Thus, the pectinolytic enzyme in addition to gelatine was applied as off-colour and off-turbidity composites to assess the clarity and colour intensity of Iranian date syrup using response surface methodology. The interaction of the two substances exhibited a significant improvement in decolourisation and purification, by decreasing turbidity and darkness intensity, of the enzymatically pre-treated syrups [19].

Moreover, the use of pectinase and cellulase as hydrolytic enzymes in the processing of date syrup was found to be effective in enhancing sensory attributes of enzyme-treated syrups. In addition, a lower turbidity and lighter date-syrup samples were obtained on the basis of a mixture containing 50 U of pectinase and 5 U of cellulase. Consequently, this enzymatic clarification was suggested as an important multi-unit operation to manufacture date syrup with high commercial value [26].

In similar work, preparations of pectinase and cellulase at concentrations of 0.5, 1.0 and 2.0% were tested on date syrup obtained from the ‘Birhi’ and ‘Safri’ cultivars. The results showed an important recovery of total soluble solids in syrup samples treated with enzymes compared to treatments without enzymes [27]. However, the enzymatic processing based on pectinase and cellulase indicated a decrease in phenolic (based mainly on p-Coumaric acid, catechin, vanillic acid, caffeic acid, and ferulic acid) and flavonoid concentrations, as well as in antioxidant activity [28].

The enzymatic clarification of date syrup represents a critical commercial compromise between sensory attributes and nutritional retention. While enzymatic treatment using specific biocatalysts like pectinases and cellulases effectively hydrolyses complex cell-wall polysaccharides to eliminate colloids and reduce viscosity, it simultaneously reduces the concentration of polyphenols and flavonoids. This loss is traditionally accepted because visual clarity, rheology, and storage stability orient consumer preferences [29,30].

### 2.3. Innovative Extraction Approaches

Recently, emergent, novel, and ecofriendly technologies were applied whether as a pre-treatment or as a critical extraction process to recover key functional and bioactive compounds in date syrup and other date palm by-products [30].

(a) Ultrasound-assisted extraction (ULASE)

**Figure 3:**
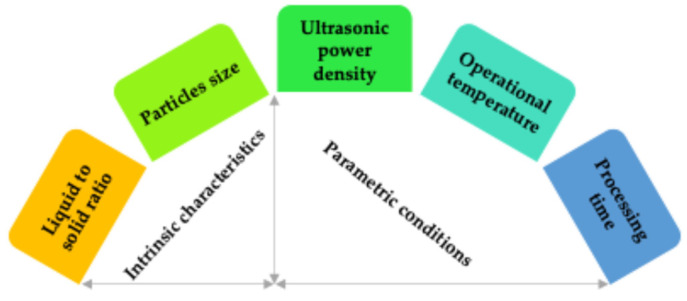
Analytical factors affecting ultrasound-assisted extraction for date syrup.

ULASE is known as a non-thermal technique used for the extraction of several bioactive constituents by complex mechanisms that involve deforming, disrupting, and breaking structural tissues and cell walls. Such mechanisms result in the increase in diffusion capacities of cell membranes, more accessibility to cell content, improvement in molecule recovery, and a high extraction rate [31]. For the specific case of date syrup fruit, ULASE was described as a promising, efficient, and cost-effective technique for the extraction of nutrients and essential physicochemical compounds from date fruit [32]. In practice, this technique consists of filling an ultrasonic instrument with date syrup and applying a defined ultrasonic frequency under specific temperature and processing time. To optimise the yield of this extraction, various factors were indicated as strong parameters to take into account while designing an ultrasound scheme for sugar concentration in date syrup [33,34,35]. Figure 3 briefly reports these factors.

In a recent investigation, ULASE was carried out on date palm powder at optimised conditions of temperature (60 °C), processing time (30 min), and extraction ratio (7.6 mL/g: liquid to solid) providing a sugar extraction yield of about 81%. This optimised ultrasound extraction demonstrated promising thermal stability and improved mass transfer diffusion [33]. Another work focused on assessing the effect of combining natural deep eutectic solvents (NDES) and ULASE in increasing yield extraction and profile of sugar extracts. Compared to conventional solvents such as water, the use of NDES based on a mixture of choline chloride–citric acid–water allowed for a high nutritive sugars recovery in addition to maintaining most bioactive compounds [34]. Ultrasonication was also applied as a preservative method to ‘Khalas’ cultivar-based syrup. The process consisted of treating syrup at a constant temperature of about 56 °C at 20 kHz during 20, 30, and 40 min. Based on pH and colour changes, the predicted shelf life of sonicated syrup at 40 min was the highest compared to unprocessed and thermally processed syrup. Furthermore, the sensory profile (sweetness, appearance, odour, and overall preference) of syrup samples treated for 30 and 40 min showed acceptable properties up to 3 weeks of cold storage [35]. Previously, it was confirmed that ULASE remained an effective green technique for date syrup extraction compared to microwave, enzymatic, magnetic stirring, and water path extraction [36].

Recent advancements in sustainable extraction technologies prioritize water-based binary natural eutectic solvents (aquoNDESs) and ultrasound-assisted extraction to enhance mass transfer from food matrices. Contemporary approaches integrate artificial neural networks with life cycle assessments and techno-economic assessments to bridge laboratory-scale efficiency with industrial applications [37,38].

(b) Vacuum membrane distillation

At mild–high extraction temperatures (50–70 °C), the liberation of HydroxyMethyl Furfural (HMF) as well as fructose thermal degradation may occur and negatively impact the quality of date syrup. To mitigate these undesired reactions, date syrup was processed for the first time at a low temperature of 28 °C by applying the Vacuum Membrane Distillation at a feed flowrate of 28 litre/hour and a pressure of 4 mbar [39]. This concentration was carried out using microporous and hydrophobic flat polypropylene membranes with different porosities and thicknesses. According to the preliminary results of this work, it was concluded that the effectiveness of this extraction can be improved by the use of membranes with high flux abilities at low pressure [39]. An ultrafiltration pilot using a CARBOSEP membrane made of zirconium oxide with carbon support was also suggested for the clarification of date palm sap syrups obtained from a Tunisian palm grove [40]. As limitations of these membranous techniques, it was pointed out that membrane fouling, limited durability, and high processing costs, as well as the low concentration threshold (especially for reverse osmosis), are the main constraints that reduce the industrial use of this extraction technology [41,42].

(c) Ohmic heating and other thermal extraction technologies

Recently, new systems aiming to accelerate the process of date syrup extraction and increase the productivity at an industrial scale were recommended [22,23,43]. Consequently, an electro-thermal extraction process was suggested to concentrate sugars in date syrup by using solar energy and hydraulic pressure. Thus, the heating “of date syrup medium” was ensured by solar thermal energy while hydraulic pistons were employed to squeeze date fruit at two pressure levels of 6 and 7 bar. In comparison with traditional extraction, the process duration and the productivity were increased by 38% and 36.7% respectively when “the high-pressure level” was applied and “the solar water bath” was used to catalyse the extraction [22]. For sensorial properties, no significant differences were found between the traditional and improved extraction methods that allowed for the production of ‘Khalas’ date syrup with good appearance, colour, and viscosity [22].

Meanwhile, hydraulic pressure (as a mechanical factor) and moderate temperature (as a thermal factor) were assessed jointly in the improvement of date syrup extraction. The process optimisation was performed by exploring response surface methodology and central composite design tools. This approach revealed a decrease in acidity and an increase in key bioactive compounds such as sugars, pectin, ash, and proteins. In addition, the extraction efficiency and yield were optimised at the highest ranges when the parametric conditions of processing were 60 °C for temperature, 8.83 bar for pressure, and 6 h for the process time [23].

Based on a similar concept, an innovative approach based on ohmic heating technology was carried out on the ‘Sukkari’ cultivar to optimise extraction yield and investigate the potential effects on physicochemical and sensory characteristics. Thus, an ohmic system (electrode equipped cylinder) was designed, fed with a mixture of date paste and water, and heated for 10–12 min at different electric field strengths of 9, 10, and 11 Volts per cm. Therefore, the prototyped device allowed for the recuperation of the filtered date juice in a connected cylinder and its evaporation until the endpoint of 72 °Bx. The obtained results indicated a higher yield of sugars recovery for the electric field of 11.5 V/cm, and sensory attributes were highly scored for the ohmic-based syrup compared to the conventional heating method. For physicochemical properties, no clear tendency was confirmed oppositely to extraction cost that was lower for the case of ohmic extraction [43]. Moreover, it was found that the thermal concentration of date palm sap syrup impacted the Maillard reaction products (namely 5-hydroxymethyl-2 furaldehyde and 2-furaldehyde) that increased when the extraction temperature reached 100 °C, confirming high reactivity between some reducing sugars and amino acids. Nonetheless, it was shown that this extraction process significantly improved the antioxidant activity and phenolic compounds [40].

Overall, the extraction technologies used for date syrup extraction differ in their efficiency, processing conditions, and product quality enhancement. A comparative summary is presented in Table 2.

## 3. Characterisation and Quality Properties

### 3.1. Compositional and Functional Characteristics

Date fruit syrup is a complex liquid rich in carbohydrates (glucose and fructose mainly), minerals (potassium, magnesium, iron, etc.), vitamins (riboflavin, thiamine, etc.), and unsaturated fatty acids (especially oleic and linoleic acids) [12]. The concentration of carbohydrates in syrup is highly impacted by the extraction and concentration process as well as by the initial content of sugars in date palm cultivars. The main three sugars in date syrup are glucose, fructose, and sucrose. Glucose and fructose ranged between 21.8–29.8 and 23.3–32.5 g/100 g, respectively, while sucrose was detected at a lower range of 3.28–14.7% [44]. In another study, the concentration of reducing sugars (glucose and fructose) ranged between 62–67.85 g/100 g of fresh weight (FW), whereas the amount of non-reducing sugars was in the range of 2.3–3.5 g/100 g of FW [45]. The lower content in terms of sucrose in date syrup was confirmed in a recent study reporting only 2.15 g/100 g for date syrup prepared using an open heating extraction [46].

Proteins are moderately abundant in date palm syrup with a range of 1.43 to 4.62 g/100 g of DW [44,47] indicating a possible effect of cultivar and extraction approaches that may increase the rate of protein denaturation during thermal processes.

Date syrups are also a good source of many minerals such as potassium, sodium, and magnesium with a respective value of 1000, 90.5, 52 mg/100 g of FW [45]. Thus, the average concentration of ash in date syrup was approximatively ranged between 1.23 and 1.76 for three Omani date syrups: ‘Mabseeli’, ‘Um-sellah’, and ‘Shahal’ [48]; 2.18 for the ‘Kebkaab’ Iranian cultivar [47]; and 2.05–2.97 g/100 g for the ‘Barhi’ Saudi cultivar [46]. Other major components of date syrup are organic acids recognised as enhancers of sensorial characteristics. A chromatographic analysis showed that syrup extracted from Moroccan date cultivars is rich with acetic, succinic, citric, malic, and oxalic acids. Among these compounds, malic and succinic acids were the most predominant and representative with a range of 671–1003 and 209–627 mg/100 g of dry weight [44]. The dietary fibres content (DFC) of some date fruit syrup was also investigated and exhibited significantly lower concentration compared to fresh date fruit before processing [49]. Accordingly, the DFC was 50- to 180-fold higher in date fruit powder [48] than the range of 0.01–0.29 g/100 g of FW for processed date syrup.

Regarding polyphenols, available literature indicated different ranges for phenolic compounds depending on the extraction method of the syrup, chromatographic and separation analysis, intrinsic attributes of the date cultivar employed for syrup production, and also the unit of expressing the result (such as dry or fresh weight). So, a comparative study was carried out to elucidate the concentration of polyphenols in date syrups processed from three cultivars cultivated in different regions, suggesting a large range of 434–769 mg GAE/100 g [50]. Similar amounts of 618 and 605 mg GAE/100 g were obtained for ‘Tamesrit’ and ‘Khadrawi’ date syrups, respectively [51,52]. Meanwhile, a lower range of total phenolic compounds (TPC) was indicated for ‘Barhi’-based syrup processed according to an open heating extraction [46]. Further ranges of TPC suggested for other date syrups are given in Table 3.

The exact profiling of phenolic compounds in terms of phenolic acids was not approached in the available literature, since its extent was limited only to the determination of TPC and in some cases “flavonoids”, “tannins”, and “flavonols”. For “flavonoids”, a range of 7.37–40.5 mg EQ/100 g FW was indicated [20,47,52], while “tannins” were cited in limited studies that suggested non-similarity in the concentration of this phenolic compound (Table 3). Overall, the phytochemical composition shown in Table 3 clearly indicates that date syrup is a substantially rich matrix with numerous components that may have a strong impact on processing behaviour and antioxidant, biological and antimicrobial properties, as well as health-promoting effects. The abundancy of these bioactive compounds and their interaction may also lead to the release of some undesirable components (e.g., Hydroxymethylfurfural) and contribute to unsuitable reactions such as Maillard reactions.

### 3.2. Antioxidant and Antimicrobial Potentials

Frequently, antioxidants were linked to the prevention of several diseases and demonstrated large antibacterial activity and positive effects against antioxidative stress. Date palm syrup was reported in many studies as an effective and good source of natural antioxidants and phenolic compounds (Table 2) that might act according to various mechanisms and chemical reactions: chelation of iron, removing free radicals, reducing hydrogen peroxide, etc. [50,51,52].

The in vitro antioxidative activity of three date syrups was investigated using seven methods, mainly Thiobarbituric acid-reactive substances (TBARS), the DPPH free radical scavenging assay, the nitric oxide (NO) scavenging activity, etc. Generally, all analysed syrups showed high to very high antioxidant activity that could reach 97.4% of inhibition when the TBARS was used for date fruit processed at the not-yet-completed ripening stage. Also, the antioxidant potential was found to be positively correlated with the total phenolic content and total flavonoids content [50]. Moreover, the antioxidant activity of four Algerian date syrups exhibited a wide inhibition range of 48.2–97% based on the DPPH test [51]. Compared to date concentrate and date liquid sugar, the DPPH Radical Scavenging Activity of date syrup was obtained from the ‘Kebkaab’ cultivar processed using hot water and vacuum evaporation [47]. Another recent study suggested higher DPPH values for syrups prepared by enzymatic extraction coupled with open heating evaporation when compared to those obtained under vacuum [46].

The extraction process has been indicated as a key element in improving (or not) the phenolic content recovery and increasing the antioxidant potentialities, especially for low–mild temperature extraction processes as well as enzymatic extraction based on cellulase and pectinase enzymes. Thus, it was confirmed that enzyme-based extraction exhibited a higher total polyphenolic content compared to identical extraction without enzymes [46]. Accordingly, date syrup polyphenols (DSP) are considered as active vectors directly involved in inducing oxidative stress in bacteria as a result of hydrogen peroxide generation. In this context, it was established that DSP stopped the proliferation of bacteria at low concentrations (of 20–30 mg/mL) and acted as bacteriostatic agents against *Escherichia coli* and *Staphylococcus aureus*. A study also highlighted a critical cause–effect relation between the polyphenol concentration in date syrup and the direct effect on antioxidant activity and bacteria inhibition [52]. Earlier, the antimicrobial effect of a traditional Tunisian date syrup was qualified as highly effective against a range of pathogenic strains such as *Staphylococcus* epidermidis and *Bacillus cereus* [54].

### 3.3. Health-Promoting Properties

Date fruit and related by-products recently gained specific importance because they contain sugars, minerals, dietary fibre, and phenolic compounds that have been investigated for various biological activities including antidiabetic, cytotoxic, Angiotensin-Converting Enzyme inhibitory activity, lowering hypercholesterolemia, bone-stimulation activities, etc. [54,55,56].

Date syrup is known as a sweet beverage used in traditional ways to act as a natural matrix and may contribute to the prevention and treatment of several diseases. Likewise, the richness of date syrup in terms of polyphenols contributed to reducing the inflammation risks and attenuating the angiogenesis process according to a complex mechanism that mainly involves the inhibition of endothelial invasion [57]. Its consumption has also been related and associated with an improvement in liver function, a reduction in DNA damage, and lower serum inflammatory cytokine levels in mice following ionising radiation treatment, hinting at a potential radioprotective effect in cancer scenarios where radiotherapy is prescribed [58]. The valorisation of date syrup in a fermented formulation leads to a confirmed cytotoxicity effect against some cancer cell lines (e.g., Caco2 and MCF-7), emphasising the potential biosynthesis and release of functional and secondary metabolites [59]. The bio-fermentation based on date syrup fortification increased the Bifidobacterial concentration and proved the co-effect of date syrup oligosaccharides as prebiotics in enhancing probiotic characteristics and raising potential positive effects in the digestion process [60]. Recently, it was reported that an isotonic drink produced from ‘Ajwa’ date syrup offered significant health benefits by substituting for artificial sweeteners [61].

## 4. Main Applications in Food Industry

**Figure 4:**
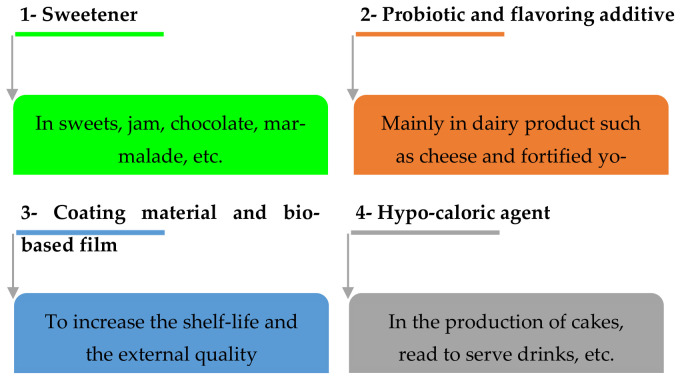
Key applications of date syrup in the food industry.

The food industry (FI) has shifted gradually to produce more functional products with high nutritional properties, appreciable sensory value, and nutraceutical attributes meeting the increasing needs of consumers. Given its qualitative characteristics, date syrup is largely employed in several branches of FI mainly as a sweetener as well as a fortifying agent in dairy product, coating material, etc. (Figure 4).

### 4.1. Dairy Products Fortified with Date Syrup

Currently, date syrup is widely used in the dairy industry to process and enhance dairy products such as cheese, desserts, and yogurts. Thus, a novel processed cheese with health benefits was produced with different proportions of date syrup (15, 20, and 25%). This fortification suggested an improvement in sensory quality as well as a general increase in physicochemical properties that gradually increased upon the increase in syrup concentration [62].

Furthermore, a probiotic fermented camel milk was supplemented by 6 and 8% of a commercial date syrup and assessed for its biochemical, microbiological, and sensory attributes. The 8%–based fermented milk presented the highest sensory rates and nutritional value, ensuring the manufacture of natural, sweet, symbiotic, and healthy flavoured and fermented camel milk [60]. In the same range of studies, the “Zabady” fermented dairy product with 2% of the ‘Saidy’ cultivar syrup improved the organoleptic and functional characteristics based on all essential amino acids besides histidine, lysine, threonine, and leucine + isoleucine [45]. Similarly, “Laban” fermented milk incorporated with six specific concentrations of date syrup ranging from 2.5 to 15% showed the opposite effect on both physicochemical and microbiological parameters. Thus, total solids, sugars, proteins, ash, and pH were shown to exhibit a progressive increase in contrast to a decrease in fatty content, casein, lactose, acidity, and microbial load. In addition to its role as a flavoured component, date syrup also acted as a shelf-life enhancer allowing for the preservation of nutritional, microbial, and sensory qualities during cold storage [63].

Moreover, a multi-unit operation process was adopted to develop a flavoured milk drink with ‘Sukkary’ and ‘Khalas’ date syrup based on full-fat cow and camel milk. Among four percentages of 5, 10, 15, 20% of dibs (the local name for date syrup), the 10% syrup-based milk drink with ‘Khalas’ syrup was highly appreciated. However, all formulations were suggested as natural, distinctive, and nutritionally rich milk drinks that can be easily commercialised as new dairy products [56]. In the category of dairy products, a flavoured dairy dessert was processed by mixing 14% of date syrup and 2% of date powder after an optimisation process using D-optimal mixture design. According to its good acceptability, the flavoured dessert was qualified as a new dairy formulation that can be used to substitute commercial dairy desserts with white sugars and artificial additives [64]. A comparative study investigated the consumer perception of flavoured yogurt, with 2% and 5% of date syrup, made from pasteurised and sterilised cow milk. Compared to unfortified and pasteurised yogurts, the 5% fortified yogurts prepared with sterilised milk were more appreciated in terms of flavour and texture properties [65].

In addition to elucidating the flavouring effect of adding date syrups to dairy products, the assessment of sucrose substitution in fermented milk was carried out by the use of Gamma irradiated date syrup. This substitution was effective since it improved the sugar content, polyphenol profile, antioxidant activity, and proteins, as well as sensory attributes [66].

### 4.2. Energetic and Isotonic Sports Drinks

This category of beverages is developed for athletes on the basis of specific components including carbohydrates, minerals, etc. Frequently, synthetic ingredients are used to process these isotonic sport drinks and occasionally natural components are chosen as raw inputs before processing [61,67]. Accordingly, three novel liquid formulations based on ‘Ajwa’ pulp (20, 25, and 30%) and deionised water (80, 75, and 70%) were suggested as an alternative for conventional drinks found in the markets. The qualitative analysis, based on biochemical, sensory, and microbiological attributes, in addition to storage ability, revealed that the “Ajwa Soft Drink” with 25% of date pulp was found to be more nutritious, appreciable, and stable [61]. Another similar work was interested in developing a “ready to serve drink (RTSD)” by diluting date palm juice at different levels (10%, 20%, 30%, and 40%) in distilled water. The 30% based RTSD was highly appreciated and secured a low microbial load and high acceptability during storage [68].

### 4.3. Formulations-Based Date Syrup: Natural Jellies, Chocolate, Cake, Yogurts, and Ice Cream

The substitution and replacement of white sugars in food formulations became currently a society issue for several consumer communities paying more attention and consideration to healthier lifestyles and dietary patterns. Thus, minimising sugar intakes directly impacts the obesity and overweight risk as well as diabetic disorders [69]. In this perspective, date syrup was used as a natural source of sugars in the manufacture of functional and healthy products, as it was mentioned before in the case of flavoured yogurts and energetic drinks. Another niche of interest in the food industry field may be promising, according to a few studies that investigated new prospects for processing sponge cake, fortified chocolate, ice cream, jellies, etc. In Table 4, a summarised overview of date syrup-based formulations is reported.

In addition to the above-mentioned food matrices, a natural soluble sweetener was developed by employing a complex extraction and flotation processing scheme [75]. Another interesting application is the use of date syrup as a replacement for granulated sugar, as its lower glycaemic index (date syrup has a moderate blood sugar impact, while granulated sugar has a high impact) makes it a good solution for the development of foods with a lesser impact on blood sugar levels and allowing for better appetite control. An example of this has been reported by Famuwagun et al. [76] who found that replacing granulated sugar with date syrup in bread resulted in a 10% to 20% reduction in the overall predicted glycaemic index of the bread samples, shifting the final product from a high glycaemic index (>70) to a medium one. Moreover, these samples were also reported as having a significant inhibitory impact upon a-amylase and a-glucosidase enzymes, two carbohydrate-hydrolysing enzymes whose inhibition has been associated with positive management outcomes for the management of diabetes.

Moreover, date syrup was recommended as a natural additive for the formulation of new, green, and non-synthetic perfumed soaps since it improved the antibacterial and antioxidant properties [77].

### 4.4. Edible Packaging and Coating

Advanced packaging techniques such as edible coatings are among the innovative and sustainable solutions for enhancing food quality and safety, extending shelf life, and reducing environmental impact [78,79]. In this context, date syrup presents itself as an interesting multifunctional ingredient, whose composition provides a unique base to improve both the structural and functional performance of coating systems. Fructose and glucose, some of the major sugars found in date syrup, have been described as functioning as a plasticizer in starch-based edible films, enhancing flexibility and reducing brittleness due to their capacity to disrupt intermolecular interactions between the polymer chains which results in a higher chain mobility. Moreover, some authors reported that fructose incorporation reduced the water absorption capacity of the film as well making it a more effective moisture barrier while keeping the film’s water activity low to help inhibit microbial growth [80,81]. An example of this application is the combined preparation of date syrup and starch which was employed as a protective packaging film and proved efficiency in terms of increasing the thermal stability and improving mechanical properties and optimal absorption of ultraviolet rays [82]. Also, a date syrup-based gel was formulated (after the high-pressure homogenisation of syrup) and tested in the gelatinisation of corn starch. The obtained gel enclosed nanoparticles that contributed significantly to reducing the water activity and pasting properties of corn starch [83]. A parallel investigation was carried out on sweet potato starch (SPS) and confirmed a strengthening effect of date syrup in positively impacting the pasting, textural, and dynamic rheological properties of SPS compared to glucose/fructose blends [84]. Similarly, the structural and rheological properties of Succinoglycan biogums were improved when date syrup was used instead of sucrose medium [85]. Moreover, the presence of a significant mineral content in date syrup (like potassium, calcium, sodium and magnesium) can also play a role in the modulation of the films and the coatings’ mechanical strength as they may cross-link with the biopolymers (e.g., alginate or whey protein) used as the base network [86].

Beyond its potential to improve the structural/mechanical properties, the incorporation of date syrup into the edible coatings can also bring additional advantages. One of the most relevant stems from their relatively high phenolic content, which encompasses flavonoids and tannins, compounds known for their antimicrobial and antioxidant and antioxidant potential. This means that their incorporation into coating systems can impart them with the capacity to inhibit microbial growth as well as to mitigate the oxidative degradation that occurs during storage [52,53,54]. The presence of melanoidins can also support additional functionality in the coatings. Reported as being capable of absorbing UV light, the incorporation of date syrup melanoidins into the coatings can also protect against photodegradation, further improving the performance of the coatings [87,88]. In fact, it has been reported that date palm syrup incorporation into starch films results in films with a significantly higher UV-absorption capacity. Moreover, it is interesting to note that with the increase in UV exposure time, there was also an improvement in the UV absorption capacity of the films, hinting at the formation of additional light-absorbing chromophoric structures [82].

Overall, the incorporation of date syrup into edible coating formulations presents itself as an interesting possibility for the development of multifunctional packaging systems as it combines structural re-enforcement with antimicrobial and antioxidant functionality which enhances the performance of the coatings and extends shelf life while promoting more sustainable food systems. Furthermore, the image analysis and machine learning approaches offer a promising tool for assessing the effectiveness of date syrup-enriched edible coatings. The use of non-destructive techniques based on images features [89] could enable rapid and objective assessment of the changes in colour, texture, and surface characteristics of coated matrices during storage.

## 5. Conclusions and Future Prospects

This comprehensive review described the high functional, nutritional, and dietary prospects for date syrup and suggested it as a promising beverage that can be consumed alone or incorporated into other food formulations for a healthy lifestyle. Furthermore, it was pointed out that this natural sweetener (date syrup) constitutes a strong alternative to sugars and artificial sweeteners and additives.

Nevertheless, further metabolomic investigations are needed to achieve a more comprehensive profiling of bioactive and multifunctional compounds and substances. Moreover, although techniques for the extraction and purification of date syrup are gaining more interest in the scientific community for an effective application in the food industry, additional deep research could be carried out to address challenges and mitigate some technological aspects such as the Maillard reactions, darkening phenomena, etc. Furthermore, future studies should focus on developing standardised industrial date syrup extraction protocols, the optimisation of processing conditions, the scalability of novel extraction methods, and conducting technico-economic assessments of emerging extraction technologies to facilitate their commercial implementation.

## Figures and Tables

**Table 2:** Comparative summary between date syrup extraction techniques.

Extraction Technique	Yield	Processing Conditions	Scalability	Colour and Turbidity	HMF Formation	Bioactive Molecule Retention
Traditional	Moderate	High temperature and long time	High	Poor	High	Low
Enzymatic	Moderate to High	Mild	Moderate	Excellent	Low	Moderate
Ultrasound-assisted	High	Mild and short time	Promising	Good	Very low	High
Membrane-based	Moderate	Low temperature	Moderate	Excellent	Very low	High
Ohmic heating	High	Rapid volumetric heating	Promising	Good	Lower than conventional heating	Moderate to High

**Table 3:** Functional compounds of date syrup processed from several cultivars.

Date Palm Cultivar	Extraction Method	Phenolic Content (mg GAE/100 g)	Antioxidant Activity	Tannins (TN) or Ash (AS) in g/100 g	Dietary Fibres (DF) or Flavonoids (FV)	Carbohydrates (g/100 g)	Proteins (g/100 g)	Reference
Saidy	Thermo-evaporation	173	ND	AS = 1.84	DF = 0.35	67.9	1.75	[45]
Barhi	Open heating	5360	5.68 × 10^−2^ (micro mol TE/100 mL)	AS = 2.97	DF = 0.98 (g/100 g)	61.5	0.81	[46]
Bouslikhene	Heating and pressing	5390	562.8	AS = 3.19	ND	68.7	2.34	[44,53]
Bousthammi		5840	760.6	AS = 3.82	ND	75	2.69	
Jihel		6700	382 microgram/ml	AS = 2.48	ND	61.8	4.07	
Khadrawi	Not specified	605.1	Radical scavenging (%) 87.2	TN = 357.4	FV = 40.5 mg/100 g	ND	ND	[52]
Rotab (Yemen)	Thermo-evaporation	769.6	94.0	TN = 554.0	ND	ND	ND	[50]
Tamar (Iraq)		434.3	91.1	TN = 310.5	ND	ND	ND	
Tamar (Saudi Arabia)		600.3	94.3	TN = 372.7	ND	ND	ND	
Mabseeli	Thermo-evaporation	162	84 micromoles of Trolox equivalents (TE) per gram	AS = 1.41	DF = 0.18	74.2	0.95	[48]
Um-sellah		141	174	AS = 1.76	DF = 0.29	73.6	0.95	
Shahal		96	106	AS = 1.23	DF = 0.01	62.7	1.09	
Tamesrit	Conventional heating at 65 °C	612	Inhibitory concentration (mg/mL) = 0.06	TN = 100 mg Catechin equivalent/100 g	ND	74 °Bx	ND	[51]
Deglet Nour	Conventional heating at 100 °C	442	ND	ND	ND	81	ND	[49]
Kentichi		402	ND	ND	ND	82	ND	
Allig		397	ND	ND	ND	85.5	ND	
Kebkaab	Heating and enzymatic	453	0.18 (IC50 TBHQ/IC50 Sample)	ND	ND	27.7 of Glucose and 25.2 of Fructose	1.43	[47]
Ghars	Traditional	768	2.09 DPPH (IC50 mg/mL)		FV = 7.37 mg EQ/100 g FW	62.4	2.33	[20]

ND: Not Determined; TBHQ: Tertiary Butyl Hydroquinone; IC: Inhibitory Concentration; DPPH: 2,2-diphenyl-1-picrylhydrazyl.

**Table 4:** Short overview of the main food products processed based on date syrup.

	Yogurts	Fortified Chocolate	Jelly	Ice Cream	Sponge Cake
Running technological process	Milk pasteurisation and cooling at 40 °C **↓** Adding date syrup **↓** Inoculation with bacteria culture **↓** Coagulation and fermentation **↓** Refrigeration at 5 °C **↓** Storage	Mixing ingredient (cocoa butter, cocoa powder, water) **↓** Incorporation of date syrup **↓** Double-boil heating and stirring **↓** Solidification of molten chocolate **↓** Re-heating at 32 °C **↓** Moulding	Mixture preparation: 20% of orange albedo powder in lemon juice and 80% of date syrup **↓** Cooking at 150 °C for 10 min **↓** Chilling **↓** Storage	Fresh cream, skim milk powder, sucrose, and gelatine blending **↓** Adding date syrup as sugar substitute **↓** Heating at 80 °C for 15 min **↓** Cooling at +4 °C for about 15 h **↓** Freezing and packaging	Mixing sugar (and/or date fruit syrup) with egg yolk **↓** Addition of beaten egg white **↓** Slow adding of wheat flour **↓** Mixture moulding **↓** Oven baking at 180 °C/15 min **↓** Storage
Quality improvement	Increase in sensory scoring, storage ability, and some functional compounds	Date syrup can be a promising alternative to substitute sugars and produce sugar-free chocolate	Production of date syrup-based jelly with suitable textural properties	The addition of date syrup (up to 60%) can replace sugars and improve the flavour attribute	Sponge cakes in which sugar was replaced by date syrup showed higher levels of polyphenols and antioxidant activity
Reference	[62,65]	[70,71]	[72]	[73]	[74]

## Data Availability

No new data were created or analyzed in this study. Data sharing is not applicable to this article.
